# The Effect of a Secondary Process on the Analysis of Isothermal Crystallisation Kinetics by Differential Scanning Calorimetry

**DOI:** 10.3390/polym12010019

**Published:** 2019-12-20

**Authors:** Catherine A. Kelly, James N. Hay, Richard P. Turner, Mike J. Jenkins

**Affiliations:** School of Metallurgy and Materials, University of Birmingham, Birmingham B15 2TT, UK; c.a.kelly@bham.ac.uk (C.A.K.); j.n.hay@bham.ac.uk (J.N.H.); r.p.turner@bham.ac.uk (R.P.T.)

**Keywords:** DSC, Avrami, poly(3-hydroxybutyrate-co-3-hydroxyvalerate), secondary crystallisation, kinetics

## Abstract

This paper demonstrates the application of a modified Avrami equation in the analysis of crystallisation curves obtained using differential scanning calorimetry (DSC). The model incorporates a square root of time dependence of the secondary process into the conventional Avrami equation and, although previously validated using laser flash analysis and infrared spectroscopy, is not currently transferable to DSC. Application of the model to calorimetric data required long-duration isotherms and a series of data treatments. Once implemented, the square root of time dependence of the secondary process was once again observed. After separation of the secondary process from the primary, a mechanistic n value of 3 was obtained for the primary process. Kinetic parameters obtained from the analysis were used in the model to regenerate the fractional crystallinity curves. Comparison of the model with experimental data generated R^2^ values in excess of 0.995. Poly(3-hydroxybutyrate-co-3-hydroxyvalerate) was used as model polymer due to the prominent secondary crystallisation behaviour that this polymer is known to display.

## 1. Introduction

Poly(3-hydroxybutyrate) (PHB) and its copolymer poly(3-hydroxybutyrate-co-3-hydroxyvalerate) (PHBV) have shown promise as sustainable and biodegradable alternatives to current oil-derived, single-use plastics [1]. More recently, they have also been used as a property modifier in the 3D printing of poly(lactic acid) to enhance the mechanical properties [2]. PHB copolymers are synthesised by a range of bacteria as a means of energy storage when there is a limited supply of specific nutrients [3,4]. The polymer granules that are formed can be extracted from the cells and processed into pellets or sheets. Bacterial synthesis results in stereospecific polymers and in this case, crystallinities of 50%–80% are generated, which can render the material brittle [5,6]. In addition, the low glass transition temperature of PHB and PHBV (~4 °C) [5] allows the secondary crystallisation process to proceed at room temperature, causing progressive embrittlement [7,8]. The properties of PHBV can be influenced by the processing conditions. The mechanical properties can be improved by rapidly cooling the polymer to yield microstructure that is composed of relatively small spherulites, but to enable this, knowledge of the crystallisation kinetics is required [9]. The crystallisation kinetics of PHB and its copolymers has been investigated previously [10,11,12,13,14]; however, these analyses have only focussed on primary crystallisation and are therefore not entirely representative of the behaviour of the material, especially as secondary crystallisation occurs so readily in this polymer.

The crystallisation kinetics of a polymer are generally described by the following Avrami equation [15]:

(1) $$X_{p,t}=X_{p,\infty }\left(1-e^{-Z_{p}t^{n}}\right)$$

where *X_p,t_* is the fractional primary crystallinity; *X_p,∞_* is the final primary crystallinity; *Z_p_* is the Avrami primary crystallisation rate constant; *t* is time and *n* an integer combining values attributed to both the geometry and mechanism of nucleation.

Although this equation is widely used, it often generates non-integer n values. In addition, it does not take into account the secondary crystallisation process, which has been proven to occur during polymer crystallisation [16,17,18,19]. Numerous authors have proposed modifications to the Avrami equation to account for these issues, with the most notable being Hillier [20,21], Velisaris [17] and Hay [22].

The method developed by Hillier [20] assumes an initial constant radial growth of spherulites, followed by a first-order increase in crystallinity at time *θ*. This leads to the standard Avrami equation for the primary process (Equation (1)) and a modified version for the secondary process (Equation (2)) in which the *n* exponent is taken as 1:

(2) $$X_{s,t-\theta }=X_{s,\infty }\left[1-e^{-Z_{s}\left(t-\theta \right)}\right]$$

where *X_s,t−θ_* is the fractional crystallinity formed after time *θ*; *X_s,∞_* is the final fractional crystallinity; and *z_s_* is the secondary rate constant. These two equations can then be combined to give an expression for the total crystallinity at time *t*.

(3) $$X_{t}=X_{p,t}+\int_{0}^{t}\frac{X_{p,\theta }}{X_{p,\infty }}\frac{d}{d\theta }\left[X_{s,t-\theta }\right]d\theta$$

By inserting Equations (1) and (2) into Equation (3), the complete expression becomes:

(4) $$X_{t}=X_{p,\infty }\left[1-e^{-Z_{p}t^{n}}\right]+X_{s,\infty }z_{s}\int_{0}^{t}\left[1-e^{-z_{p}\theta^{n}}\right]\left[e^{-z_{s}\left(t-\theta \right)}\right]d\theta$$

Hillier demonstrated that this process is more effective at modelling the crystallisation of poly(decamethylene terephthalate), poly(ethylene oxide) and poly(methylene) than the standard Avrami equation but also implied that the first-order process for secondary crystallisation may be too simplified [20]. Since the publication of this model, many authors have created adaptations [23,24,25], including adding higher exponents (m) for the secondary process [23], which creates additional terms in the complete expression (Equation (5)).

(5) $$X_{t}=X_{p,t}\left[1-e^{-Z_{p}t^{n}}\right]+X_{s,\infty }z_{s}m\int_{0}^{t}\left[1-e^{-z_{p}\theta^{n}}\right]\left[{\left(t-\theta \right)}^{m-1}\right].\left[1-e^{-z_{s}{\left(t-\theta \right)}^{m}}\right]d\theta$$

Velisaris and Seferis [17] have also proposed a model to account for the secondary crystallisation process. This model is based on two distinct regions, attributed to primary and secondary crystallisation, being observed in a standard double-log Avrami plot. They postulated that these processes could occur in either parallel (Equation (6)) or series (Equation (7)), giving rise to two different models. 

Parallel:

(6) $$\frac{X_{t}}{X_{\infty }}=w_{p}\left[1-e^{-z_{p}t^{n}}\right]+w_{s}\left[1-e^{-z_{s}t^{m}}\right]$$

where *w_p_* is the weight factor for the primary process and *w_s_* is the weight factor for the secondary process and the sum of these two factors equal 1.

Series:

(7) $$\frac{X_{\infty }}{X_{t}}=\frac{w_{p}}{1-e^{-z_{p}t^{n}}}+\frac{w_{s}}{1-e^{-z_{s}t^{m}}}$$

This model was found to provide a good fit to the isothermal crystallisation of PEEK; however, non-integer n and m values were obtained [17].

More recently, Hay et al. further evaluated the secondary crystallisation kinetics of poly(ethylene terephthalate) (PET) [22] and poly(caprolactone) (PCL) [26] and derived a different approach to the previous authors. Hay noted that the models discussed above indicated a dependence of log (*X_s_,_t_*) on time; however, the opposite is seen when plotting the data [26]. Through a series of experiments on PET and PCL, Hay discovered a square root time dependence on the growth of lamella stem length following primary crystallisation [19,27]. As this is representative of secondary crystallisation, further studies on the annealing of PET following primary crystallisation were performed, which showed the square root dependence was also applicable to fractional crystallinity [18]. These results are indicative of a diffusion-controlled process. In addition, as the thickness of the lamellae is greater at the centre of the spherulite, it was postulated that secondary crystallisation occurs as soon as the lamellae has been formed and proceeds with and beyond the primary process [22]. These factors have led to the formation of a new model for the crystallisation of polymers, which has been validated using infra-red spectroscopy [22,26] and laser flash analysis [28]. 

As primary and secondary crystallisation occur concurrently, the fractional crystallinity developed at time t is the sum of both processes:

(8) $$X_{t}=X_{p,t}+X_{s,t}$$

where *X_t_*, *X_p,t_* and *X_s,t_* are the total, primary and secondary fractional crystallinities at time *t*. Further, if the primary process follows an Avrami equation, limited by a final fractional crystallinity, *X_p,∞_*, then:

(9) $$X_{p,t}=X_{p,\infty }\left(1-e^{-Z_{p}t^{n}}\right)$$

As discussed earlier, secondary crystallisation has been found to develop via thickening of the lamellae with a dependence on the square root of time [18]. Secondary crystallisation also depends on the extent of the primary process, as it cannot take place without some degree of primary crystallisation occurring first. These factors lead to the following equation to describe the secondary crystallisation process:

(10) $$X_{s,t}=X_{p,t}k_{s}t^{1/2}$$

where *k_s_* is the secondary crystallisation rate constant. Combining Equations (8)–(10) gives:

(11) $$X_{t}=X_{p,\infty }\left(1-e^{-Z_{p}t^{n}}\right)\left(1+k_{s}t^{1/2}\right)$$

Equation (11) has the correct form to explain the shape of the crystallisation time dependence in that it increases asymptotically towards *X_p,∞_*. On completion of the primary process, the crystallisation rate will depend solely on the square root of time as the term $e^{-Z_{p}t^{n}}$ becomes negligibly small. This leads to the following equation:

(12) $$X_{t}=X_{p,\infty }\left(1+k_{s}t^{1/2}\right)$$

This model has been successfully applied to a range of polymers analysed using FTIR spectroscopy [22,26,29]; however, it is not currently directly transferable to differential scanning calorimetry (DSC) isotherms. In order to consider the universality of this new approach, it is essential to extend the study to DSC analyses. To this end, the crystallisation of PHBV containing 3 wt.% valerate was measured and analysed by adopting a new procedure inherent in the assumption that the conversion incorporates both primary and secondary crystallisation. PHBV was chosen as it is well known to display secondary crystallisation over time [7,8].

## 2. Materials and Methods 

Poly(3-hydroxybutyrate-co-3-hydroxyvalerate) (PHBV) pellets containing 3 wt.% 3-hydroxyvalerate (Tianan ENMAT Y1000P) were purchased from Helian Polymers (Venlo, The Netherlands).

Plaques of the copolymer were produced by compression moulding using a Moore E1127 hydraulic hot press (George E. Moore & Sons Ltd., Birmingham, UK). Polymer pellets (8 g approx.) were placed into a mould (152 × 158 × 0.266 mm) and inserted into the press preheated to 190 °C. The material was left to melt for 5 min before a load of 10 tonnes was applied for a further 3 min. The plaques were slow cooled in the press before discs (5 mm in diameter) were cut for DSC analysis. 

A Mettler Toledo differential scanning calorimeter, DSC 1 (Mettler Toledo, Schwerzenbach, Switzerland), calibrated from the melting characteristics of indium (T_m_ 156.6 °C, ΔH_f_ 28.45 Jg^−1^) and zinc (T_m_ 419.5 °C, Δh_f_ 107.5 Jg^−1^), was used to determine the crystallisation kinetics of the copolymer. All experiments were conducted under a nitrogen flow rate of 50 cm^3^ min^−1^ and a Huber TC100 immersion cooler (Huber Kaltemaschinenbau AG, Germany) was used to aid temperature control over extended time periods up to 1000 min. The samples (9.24 mg standard deviation 1.1 mg) were weighed into 40 μL aluminium DSC pans (Mettler Toledo), capped with aluminium lids (Mettler Toledo) and sealed with a press. Samples were held at 210 °C for 2 min to melt the polymer before being cooled to the crystallisation temperature (138 to 146 °C) at 30 °C min^−1^. This rate was chosen to prevent crystallisation prior to the sample reaching the isothermal temperature, but also to minimise overshoot of the isothermal temperature. The samples were held at this temperature for up to 1000 min to allow the polymer to crystallise well beyond the end of the primary process. Three samples were analysed for each temperature to assess repeatability. A heat of fusion (∆H^0^_f_) of 146.0 Jg^−1^ has been reported for PHB [30], and since no enthalpy of fusion has been published for the copolymer containing 3 wt.% 3-hydroxyvalerate, this value has been adopted by others in analysing copolymers of PHB with low concentrations of HV [31,32,33].

## 3. Results and Discussion

**Figure 1:**
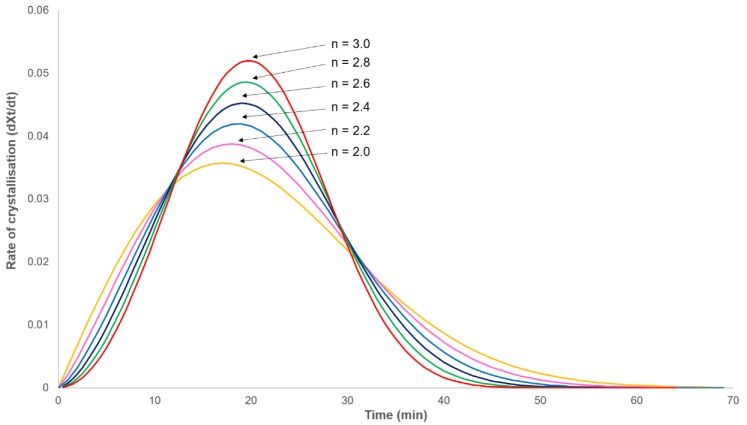
Modelled rate of crystallisation–time dependence obeying the Avrami equation (Equation (1)) with a half-life of 20 min.

In order to study the isothermal crystallisation kinetics of PHBV, heat flow was recorded as a function of time at a constant temperature in the range 138 to 146 °C for time periods greater than four times the half-life of the primary process (as estimated from the minima in the exotherm minus the induction time). This time was chosen as modelling the primary crystallisation process (Equation (1)), with an example half-life of 20 min, showing that the crystallisation rate will always fall to zero at three times the half-life when *n* = 2 or at twice the half-life when *n* = 3 (Figure 1). In light of this, it can be assumed that no further primary crystallisation will occur after this time and therefore, the secondary crystallisation process can be observed in isolation. 

**Figure 2:**
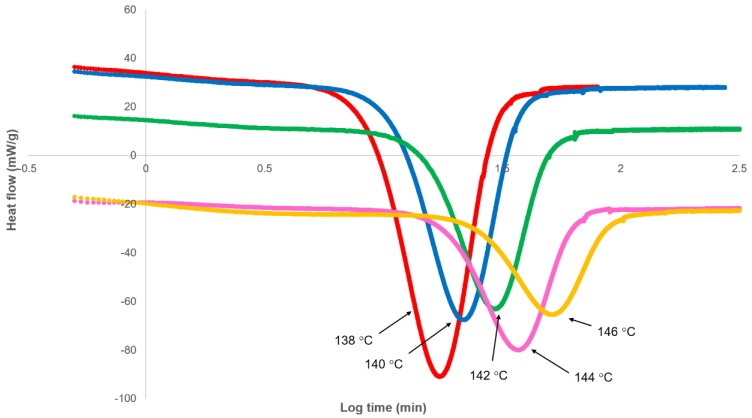
Differential scanning calorimetry (DSC) heat flow response on cooling to crystallisation temperature as a function of log experimental time.

The variation of heat flow with log time, as measured by DSC, on crystallising PHBV at various isothermal temperatures from 138 to 146 °C is shown in Figure 2. Following cooling, an initial period was observed, during which heat flow was relatively constant as the sample attained the isothermal temperature and before it decreased due to the onset of crystallisation. As shown in Figure 2, this induction period varied from 30 to 1000 s, increasing with temperature. As a consequence, experimental time was not set at the start of crystallisation, and therefore, a correction was made for this induction period (*t_i_*). In the analysis, the induction period was used as an adjustable parameter limited by a maximum value, i.e., the observable onset of crystallisation.

Prior to analysing the data, a further correction was required. Unlike polymers that display minimal secondary crystallisation, the heat flow does not return to the baseline following completion of the primary process, and therefore, the standard method of normalising the isotherm to zero heat flow via subtraction of the final heat flow (J_∞_) cannot be applied. Instead, the heat flow continues to rise at the rate of secondary crystallisation, and a different approach to determine the value of the calorimeter baseline is required. 

The heat flow-time data were restricted to greater than four times the half-life of the primary process, as estimated by the minima in the isotherm following removal of the induction time. This ensured that any changes in the heat flow were due to the secondary process alone and therefore could be calculated as:

(13) $$X_{t}=X_{p,\infty }\left(1+k_{s}{\left(t-t_{i}\right)}^{1/2}\right)$$

Differentiating this equation with respect to time gives:

(14) $$\frac{dX_{t}}{dt}=\frac{X_{p,\infty }k_{s}}{2(t-t_{i)}{}^{1/2}}$$

As the crystallinity of a material can be given as:

(15) $$X_{t}=\frac{J_{t}t}{\Delta H_{f}w}$$

where *J_t_* is the heat flow in a DSC trace and *w* is the mass of the sample, then the change in crystallinity can be given as:

(16) $$dX_{t}=X_{s,\infty }-X_{t}=\frac{\left(J_{s,\infty }-J_{t}\right)\left(dt\right)}{\Delta H_{f}w}$$

where *J_s,∞_* is equivalent to the heat flow at the end of the secondary process. Rearranging the above equation and inserting Equation (14) gives an expression for the heat flow at time *t*.

(17) $$J_{t}=-\left(\frac{\Delta H_{f}wX_{p,\infty }k_{s}}{2{\left(t-t_{i}\right)}^{1/2}}\right)+J_{s,\infty }$$

**Figure 3:**
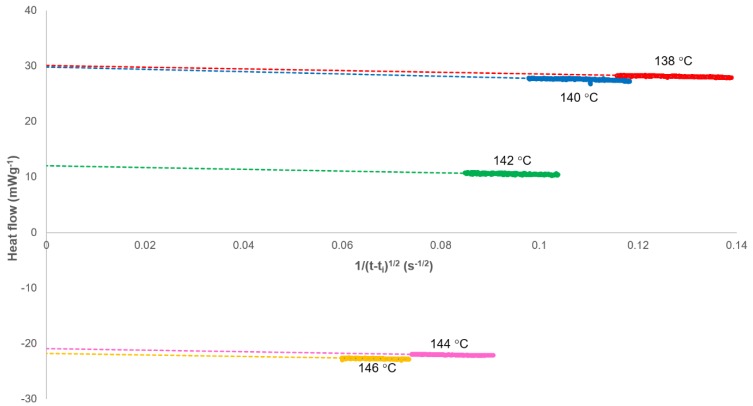
Determination of the heat flow at the end of the secondary crystallisation process (*J_s,∞_*) as calculated using Equation (17).

This equation generates a linear plot of the heat flow (*J_t_*) against (1/(*t−t_i_*)^1/2^) with an intercept at (1/(*t−t_i_*)^1/2^) = 0 equivalent to the heat flow at infinite time (*J_s,∞_*) when secondary crystallisation has ceased (Figure 3). This value was calculated for each isotherm and added to the data set before inverting the curve and integrating with time to determine the fractional crystallinity (*X_t_*). This process is equivalent to the normalisation of the heat flow in a standard Avrami calculation. 

### 3.1. Analysis of Secondary Crystallisation

**Figure 4:**
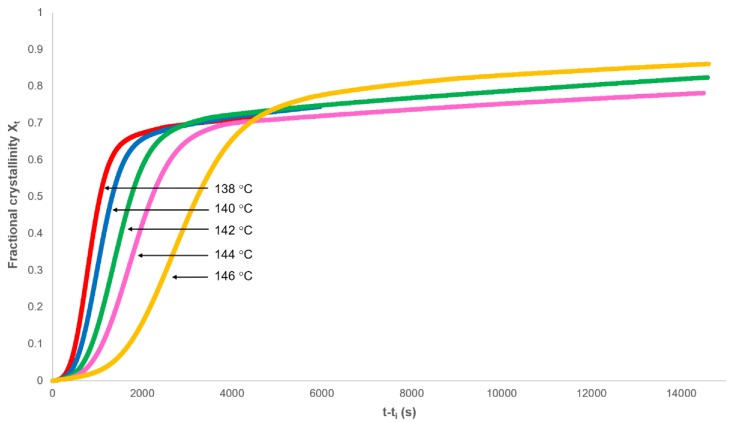
Dependence of the fractional crystallinity on time.

The isothermal portion of the heat flow against time curve was used to analyse the crystallisation of PHBV over a range of temperatures. Baseline corrections (*J_s,∞_*) were added to the heat flow data, the induction time (*t_i_*) removed, and the fractional crystallinity (*X_t_*) determined as a function of experimental time (Figure 4). Each isothermal crystallisation had the characteristics of Equation (11) in that there was an initial exponential increase in crystallinity followed by a greatly reduced dependence on time.

**Figure 5:**
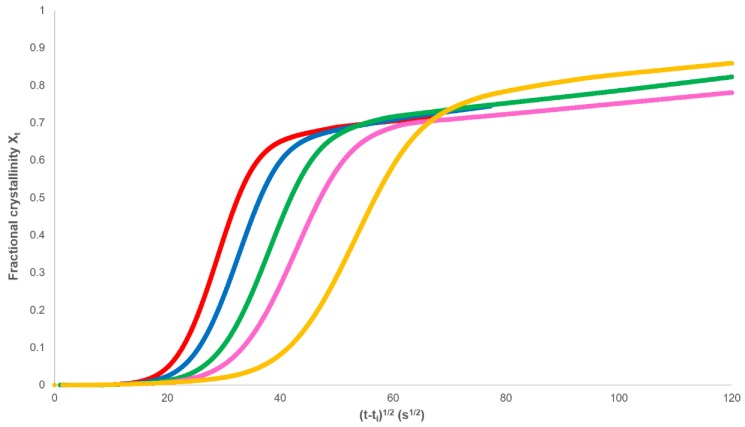
Development of fractional crystallinity with the square root of time.

The secondary rate constant *k_s_* and *X_p,∞_* can be determined from plots of *X_t_* against (*t−t_i_*)^1/2^ (Figure 5), where it can be seen that the final dependence is linear. 

**Figure 6:**
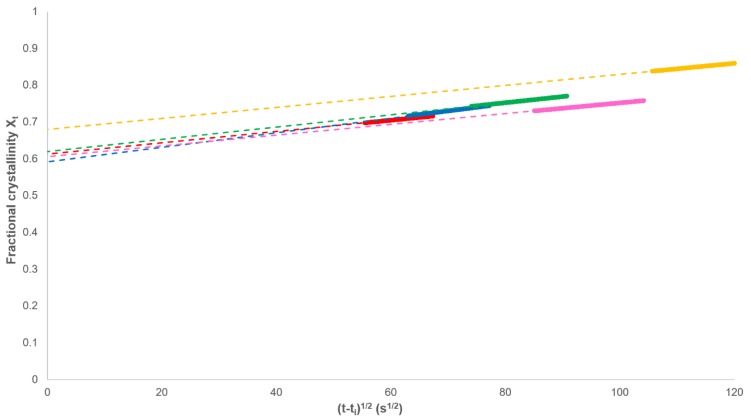
Determination of *X_p,∞_* and the secondary rate constant *k_s_* through the plotting of Equation (13).

However, as required by Equation (13), the analysis should be restricted to values of *X_t_* > *X_p,∞_* (Figure 6). In every case, a linear dependence was observed in this range and *X_p,∞_* and *k_s_* calculated from the intercept and gradient, respectively (Table 1). 

The crystallisation parameters are listed in Table 1 for both the primary and secondary processes. The limit of the primary process, *X_p.∞_*, remained consistent showing that a maximum primary crystallinity is reached independent of the temperature. The secondary rate constant (*k_s_*) also remained similar across each temperature.

### 3.2. Analysis of Primary Crystallisation

Rearranging Equation (11) gives the fractional crystallinity of the primary process alone, which follows the Avrami equation:

(18) $$\frac{X_{t}}{X_{p,\infty }\left(1+kt^{1/2}\right)}=1-e^{-Z_{p}t^{n}}$$

**Figure 7:**
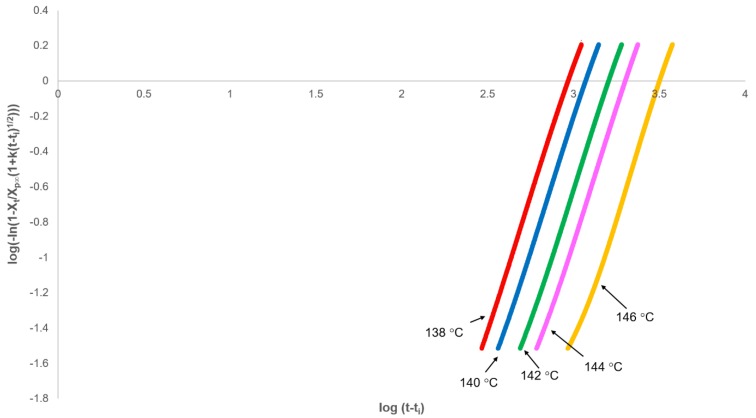
Fit of modified Avrami equation (Equation (18)) to the primary process over the fractional crystallinity range 0.03 to 0.8.

**Figure 8:**
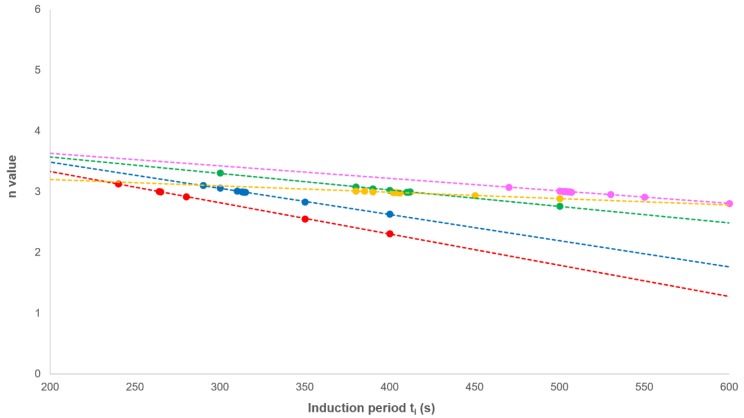
The effect of varying the induction period (*t_i_*) on the value of n.

This equation effectively removes the secondary crystallisation component from the fractional crystallinity curve and enables the primary process to be analysed from plots of $\log\left[-\ln\left[1-\left(\frac{X_{t}}{X_{p,\infty }\left(1+kt^{1/2}\right)}\right)\right]\right]$ against log(t) with slope *n* and intercept at t = 1.00 of log(*Z_p_*). In every case, non-integer *n* values were obtained; however, correcting the experimental time for an induction period (*t_i_*) using *t_i_* as an adjustable parameter over the acceptable range (200–1000 s), a value of 3.00 was observed at each crystallisation temperature (Figure 7 and Table 2). The effect of changing *t_i_* on the value of n was linear (Figure 8), which greatly reduced the number of iterations required in the analysis. 

The crystallisation rate parameters are listed in Table 2 for the primary processes where the half-life was calculated via modification of Equation (18) to give Equation (19).

(19) $$0.5=1-e^{-Z_{p}t_{0.5}^{3}}$$

Unlike the secondary rate constant (*k_s_*) (Table 1), the primary rate constant (*Z_p_*) decreased as the temperature increased, showing the process to be nucleation-controlled and therefore dependent on the degree of supercooling.

### 3.3. Applicability of the Model

**Figure 9:**
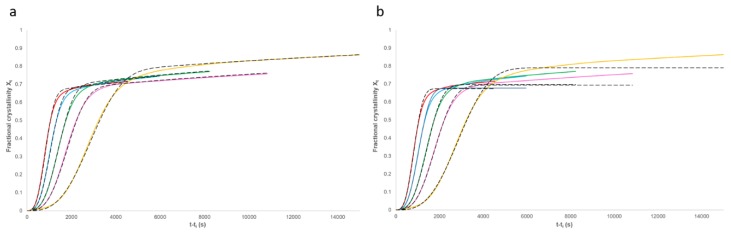
Comparison of the experimental data with (**a**) the fractional crystallinity (*X_t_*) calculated using Equation (11) and the parameters in Table 1 and Table 2 and (**b**) the standard Avrami model (Equation (1)).

Equation (11) was modelled using the rate parameters listed in Table 1 and Table 2. Comparison of these results with the experimental data (Figure 9a) shows a good agreement. A standard Avrami analysis (Equation (1)) was also performed on the data and compared with the experimental results (Figure 9b). As the end of the crystallisation process is unclear, the same baseline value and induction time were used as discussed above. Comparison of the two models shows a good agreement to a fractional crystallinity of approximately 0.6; however, above this point, a clear deviation can be seen in the Avrami model. This is because this model is not able to account for the secondary crystallisation process. In addition, non-integer n values were obtained from the standard Avrami model, which have no meaning. 

**Figure 10:**
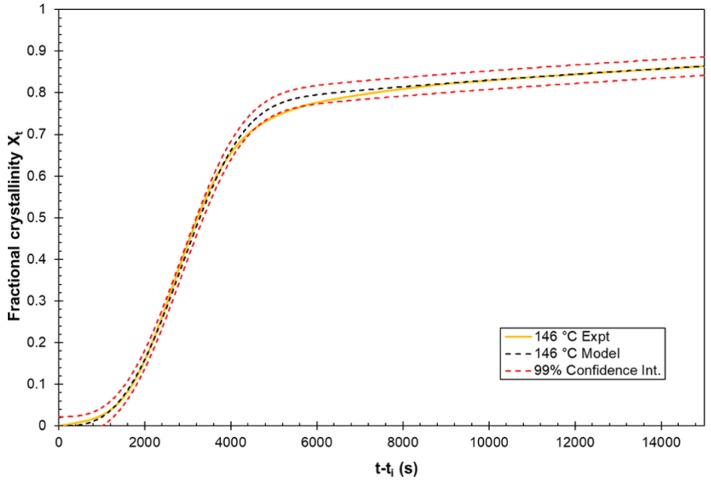
Fitting of the model to the experimental data at the 99% confidence interval.

Non-linear regression analysis using IBM SPSS software was used to measure the coefficient of determination (R^2^) fitting value for the models. This yielded results in excess of 0.995 for the proposed model at each of the five considered temperatures and only 0.896 to 0.994 for the standard Avrami model. The standard deviation was determined for the fitting of the proposed model with the experimentally measured crystallisation data for each temperature condition considered. These standard deviations values ranged from 0.00251 to 0.0074 for the best and worst fitting models, respectively. These calculated standard deviation values can then be used to generate statistical confidence intervals, with the 99% confidence interval for the 146 °C model versus the experiment shown in Figure 10.

Further statistical measures were applied to highlight the high level of precision that these models were fitting the experimental data. The Kolmogorov–Smirnov (K–S) test [34] and Kuiper’s [35] test goodness-of-fit methods were applied to compare the experimental data and the associated modelling curve at each temperature considered. A nominally “perfect” fit would return a value of zero for both these measures. For the data and models shown in Figure 9a, the goodness-of-fit measures were as given in Table 3. The additional goodness-of-fit test statistics are shown to be reasonable. As the quantity measured is fractional crystallinity with a theoretical maximum of 1.0, it becomes trivial to convert the K–S and Kuiper’s test statistics into an overall percentage error by multiplying them by 100. 

## 4. Conclusions

In a series of recent papers [22,26,28], it has been shown that the Avrami model can be modified to successfully account for a secondary crystallisation process. The modification is based on the idea that the secondary process develops with the square root of time. In this work, the model has been further developed to analyse DSC heat flow-time data generated from the isothermal crystallisation of PHBV. The rationale for the selection of PHBV is due to the prominent secondary crystallisation behaviour that this polymer is known to display. A series of DSC-specific treatments are required to enable the application of the modified Avrami equation, but when implemented, the square root of time dependence of the secondary process was once again observed. After separation of the secondary process, a mechanistic n value of 3 was obtained for the primary process. The kinetic parameters that were obtained from the analysis were used in the model to regenerate the curves showing fractional crystallinity with time, and good agreement was found. Comparison of the model with the experimental data generated R^2^ values in excess of 0.995.

## Figures and Tables

**Table 1:** Crystallisation rate parameters determined from Figure 6 and Equation (13). The results are the average and standard deviation (SD) of three repeats for each temperature.

Crystallisation Temperature/°C	Primary Limit *X_p,∞_*		Secondary Rate Constant *k_s_*/s^−1/2^ × 10^3^	
	Average	SD	Average	SD
138	0.57	0.06	4.21	2.28
140	0.60	0.04	3.49	1.66
142	0.61	0.02	2.81	1.05
144	0.61	0.10	2.65	1.21
146	0.57	0.10	4.07	1.69

**Table 2:** Crystallisation parameters for the primary crystallisation process as determined from Figure 7, Equations (18) and (19). The average and standard deviation of three repeats are reported.

Crystallisation Temperature/°C	Avrami *n* Value	−log(*Z_p_*)		Primary Half-Life/s	
		Average	SD	Average	SD
138	3.00	8.80	0.12	761	70
140	3.00	9.19	0.06	1027	46
142	3.00	9.50	0.10	1300	98
144	3.00	9.94	0.03	1824	42
146	3.00	10.41	0.10	2613	210

**Table 3:** Goodness-of-fit statistical parameters to assess how well the model predicts experimental data (all quoted to 3 s.f.).

Test Statistic	Temperature/°C				
	138	140	142	144	146
R^2^	0.999	0.999	0.999	1.000	0.999
Standard deviation (σ)	0.00591	0.00623	0.00604	0.00251	0.00740
Kolmogoro–Smirnov (K–S)	0.0213	0.0214	0.0237	0.0101	0.0263
Kuiper’s	0.0331	0.0293	0.0263	0.0211	0.0410
